# Management of pregnancy in women with cancer

**DOI:** 10.1136/ijgc-2020-001776

**Published:** 2021-02-25

**Authors:** Vera Wolters, Joosje Heimovaara, Charlotte Maggen, Elyce Cardonick, Ingrid Boere, Liesbeth Lenaerts, Frédéric Amant

**Affiliations:** 1 Department of Gynecology, Antoni van Leeuwenhoek Nederlands Kanker Instituut, Amsterdam, The Netherlands; 2 Department of Oncology, KU Leuven, Leuven, Belgium; 3 Department of Obstetrics and Gynecology, University Hospitals Leuven and Department of Oncology, KU Leuven, Leuven, Belgium; 4 Department of Obstetrics and Gynecology, Cooper University Health Care, Camden, New Jersey, USA; 5 Department of Medical Oncology, Erasmus MC Cancer Centre, Rotterdam, The Netherlands

**Keywords:** carcinoma, radiotherapy, surgery

## Abstract

As the incidence of cancer in pregnancy has been increasing in recent decades, more specialists are confronted with a complex oncologic–obstetric decision-making process. With the establishment of (inter)national registries, including the International Network on Cancer, Infertility and Pregnancy, and an increasing number of smaller cohort studies, more evidence on the management of cancer during pregnancy is available. As fetal, neonatal, and short-term pediatric outcomes after cancer treatment are reassuring, more women receive treatment during pregnancy. Prenatal treatment should adhere to standard treatment as much as possible to optimize maternal prognosis, always taking into account fetal well-being. In order to guarantee the optimal treatment for both mother and child, a multidisciplinary team of specialists with expertise should be involved. Apart from oncologic treatment, a well-considered obstetric and perinatal management plan discussed with the future parents is crucial. Results of non-invasive prenatal testing are inconclusive in women with cancer and alternatives for prenatal anomaly screening should be used. Especially in women treated with chemotherapy, serial ultrasounds are strongly recommended to follow-up fetal growth and cervical length. After birth, a neonatal assessment allows the identification of any cancer or treatment-related adverse events. In addition, placental histologic examination aims to assess the fetal risk of metastasis, especially in women with malignant melanoma or metastatic disease. Breastfeeding is discouraged when systemic treatment needs to be continued after birth. At least a 3-week interval between the last treatment and nursing is recommended to prevent any treatment-induced neonatal effects from most non-platinum chemotherapeutic agents.

## Introduction

Approximately 1 in 2000 pregnancies is complicated by cancer and the incidence has been increasing in recent decades.^1^ In countries where women still tend to postpone childbirth to a later age for socioeconomic reasons, the incidence of cancer in pregnancy will continue to increase. The implementation of non-invasive prenatal testing, which has the ability to detect preclinical cancer, is expected to further contribute to this increase.^2^ Breast cancer, cervical cancer, and melanoma are the most frequently occurring cancers in pregnancy.^3^

Oncologic management during pregnancy should be multidisciplinary; however, this continues to be a challenge. If there is a desire to preserve the pregnancy, specialists need to weigh maternal and fetal well-being and opt for the optimal oncologic treatment without significantly endangering fetal and maternal safety. In general, oncologic treatment in pregnancy should adhere to treatments used for non-pregnant patients as much as possible to conserve maternal prognosis. Besides surgery, systemic treatment, if compatible with pregnancy, plays an important role in antenatal oncologic treatment. Termination of pregnancy can be considered in the case of an aggressive or advanced-stage cancer in early pregnancy. Preterm induction of delivery in order to start oncologic treatment should be avoided where possible because of long-term morbidities of preterm children.^4^

As awareness about this complex coincidence has been increasing, research has expanded to large-scale registries.^5^ Overall, obstetric outcome has improved in the recent decades, with fewer terminations of pregnancy and fewer preterm deliveries.^5^ Changes in obstetric management have been driven by an increased knowledge of cancer in pregnancy and an overall tendency to reduce medicalization. A recent Italian monocentric study has observed a trend towards fewer elective cesareans in the last decade.^6^ This was accompanied by an increasing percentage of inductions of labor, mainly on account of the need to start or continue chemotherapy treatment.^6^ Neonatal outcome in terms of cognitive and cardiac development in the first 6 years after chemotherapy exposure is not significantly affected in comparison with children in the general population.^4 7^ Certain precautions are required as chemotherapy during pregnancy is associated with fetal growth impairment and, when administered early in pregnancy, congenital malformations.^5^

Current research has mainly focused on the feasibility and safety of oncologic treatment in women with cancer. However, reports with an obstetric point of view on this topic are scarce. Here, we describe the different aspects of obstetric and perinatal management of women with cancer and discuss fetal and neonatal effects of cancer diagnostics and treatment.

## Effects of Diagnostics on the Developing Fetus

Ionizing diagnostic imaging should be minimized during pregnancy due to the teratogenicity of radiation.^8^ Although commonly used doses are often much lower than the threshold for dose-dependent side effects of ionizing radiation (deterministic effects), exposures above this threshold have been associated with fetal death, mental retardation, malformations, and growth disturbances.^9^ The risk of stochastic carcinogenic effects of radiation depends on the gestational age during imaging and have been shown to rise with increasing dose. It is recommended that only examinations with a potential effect on the oncologic management are performed. The cumulative fetal dose of radiation should not exceed 100 mGy.^8^ The lifetime attributed cancer incidence for a radiation dose of 50 mGy is roughly estimated at 2% if the fetus is exposed after 15 weeks of gestation, but accurate quantification is impossible.^10^ In recent decades, ionizing techniques have improved and fetal radiation doses have declined. Fetal radiation exposure should be discussed with an expert radiologist and a medical physicist should conduct an a priori estimation of the fetal dose level for each pregnant individual.

Non-ionizing imaging procedures, such as ultrasound and magnetic resonance imaging (MRI) are safe techniques for staging in pregnant patients. As the fetal effects are unclear, it is recommended to administer gadolinium, a MRI contrast product, with caution to pregnant patients, outweighing the diagnostic benefit and potential fetal risks.^11^

In non-pregnant patients, fluorine-18-flurodeoxyglucose positron emission tomography integrated with computed tomography (18FDG-PET/CT) is a valuable tool for the detection and staging of different types of cancers (especially hematologic cancers and higher stages of breast cancers). However, 18FDG-PET/CT should be avoided during pregnancy because of the high dose of radiation exposure to the fetus (up to 50 mGy).^12^ Whole-body diffusion-weighted MRI has shown to be feasible in the detection of primary lesions and nodal and distant metastasis in women diagnosed with cancer during pregnancy. Furthermore, it can be used as a screening tool for underlying malignancy in the case of an aberrant non-invasive prenatal testing.^2 13^ Since there are no fetal adverse effects known, this non-ionizing imaging technique can be used safely. If indicated, pineapple juice can be used as an oral negative contrast agent for imaging optimization of the abdomen without harming the fetus.^14^

Single-day protocols for sentinel node procedures are considered safe during pregnancy.^15^ Radiopharmaceuticals can be used for these procedures when administered at low doses, not exceeding a fetal exposure of 5 mGy.^16^ The lowest possible dose of technetium can be locally injected 2 hours prior to the procedure. In such cases, approximately 90% of the technetium will be collected in the sentinel node, resulting in low systemic exposure and minimal fetal risk. Indocyanide green, a near-infrared imaging probe known for its accurate sentinel node detection and very limited placental transfer, is also widely used in pregnant patients.^17 18^ The use of blue dye for the detection of a sentinel node should be avoided since there is a small risk of an anaphylactic reaction (0.1%).^19^

## Cancer Treatment in Pregnancy

### Surgery

**Figure 1:**
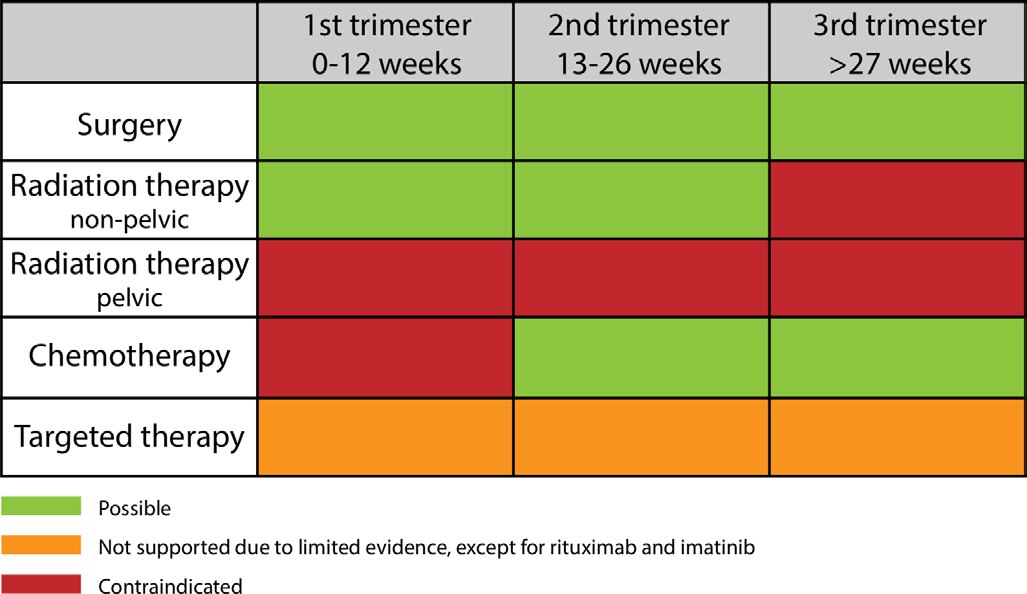
Cancer treatment per trimester.

Surgery can be performed whenever indicated, irrespective of gestational age (Figure 1).^20^ Interventions are preferably performed in the (early) second trimester, minimizing the risk of spontaneous abortion. Morbidity and pregnancy complications, such as preterm delivery and fetal distress, are more common in major abdominal and pelvic procedures due to an enlarging uterus with increased pelvic blood supply.^20^ Identical to non-cancer pregnant patients, inferior vena cava compression by the gravid uterus should be reduced by placing the patient in the left-lateral tilt position from 20 weeks of gestation onwards. Tocolytics during surgery should not be given unless uterine contractions are noted. If uterine manipulation is inevitable, post-operative administration of tocolytics can be considered for 48 hours in the late second trimester (when oxytocin receptors are present) and the third trimester of pregnancy.^21^

Laparoscopic interventions result in fewer adverse fetal and maternal events compared with laparotomy and should be chosen over open procedures when oncologically safe and if performed by an experienced surgeon, with limited operation time (90–120 min) and low intra-abdominal pressure (10–13 mmHg).^21 22^ An open entry reduces the risk of uterine perforation with a Veress needle. Pelvic lymphadenectomy can be performed until 22 weeks of gestation, both by laparotomy or laparoscopy.^21^ Later in pregnancy, the size of the uterus impedes the possibility of a complete pelvic lymph node dissection and should be avoided.

Gestational physiologic changes call for an adapted anesthesiologic approach, with extra safety surveillance to keep blood pressure and maternal oxygenation as stable as possible. If the fetus is viable (24 weeks of gestation or later, depending on local hospital policies), active management including intra-operative fetal cardiotocography monitoring and lung maturation should be discussed with the parents. Post-operative adequate pain control is important, as is intravenous hydration and thromboprophylaxis until the patient is ambulatory.

### Systemic Therapy

There are a number of factors that need to be taken into account before systemic therapy during pregnancy is administered, including physiologic changes in pregnancy, gestational age, placental passage, and the pharmacokinetic characteristics of the given drug. Physiologic changes may affect the exposure and efficacy of systemic treatments by influencing their pharmacokinetics with respect to distribution, metabolism, and excretion of drugs.

Transplacental transfer of chemotherapeutic drugs occurs by passive diffusion and is therefore based on the drug-specific molecular size, lipid solubility, protein binding, and ionization. Since most chemotherapeutic drugs have a low molecular weight and are uncharged and unbound, they can easily cross the human placenta. In baboons, fetal to maternal drug passage ranged from none for docetaxel up to a maximum concentration ratio of 0.58 for carboplatin.^23 24^

Short-term outcomes of children exposed to chemotherapy in utero are generally reassuring; however, long-term outcomes (>6 years) are unknown and the use of many targeted and hormonal therapies should be discouraged until more information on fetal safety is available.

#### Chemotherapy

Chemotherapy must be avoided in the first trimester of pregnancy to avoid interference with organogenesis (Figure 1). After 12–14 weeks of gestation, administration of most cytotoxic drugs is feasible and considered relatively safe.^25^ Standard chemotherapy regimens and doses are preferred using the actual maternal weight during pregnancy. After 35 weeks of gestation chemotherapy is usually discouraged to allow a certain window with regard to the administration scheme for maternal and fetal bone marrow recovery between the last cycle of chemotherapy and delivery. The latter should ideally be planned allowing timely resumption of postpartum chemotherapy if indicated.

#### Targeted Therapy and Immunotherapy

There are limited preclinical and clinical data on the use of targeted therapy in humans.^26^ Targeted therapy is a collective of drugs with different mechanisms of action and characteristics with implications for their use in pregnancy. First, placental passage depends on the class of drugs and their size: large molecules, for example, monoclonal antibodies (such as trastuzumab, rituximab), require an active transport via the placenta, which is present starting from the 14th week of gestation. In contrast, small molecules, such as tyrosine kinase inhibitors, can cross the placenta throughout the whole pregnancy. Targeted therapy is aimed at specific tumor-related features, some of which also play integral physiologic roles in fetal development. Consequently, these therapies may lead to an increased risk of fetal morbidity and pregnancy complications, depending on their role in fetal development.

Lambertini et al^26^ have published a comprehensive review on targeted therapy during pregnancy. For the treatment of B cell malignancies, rituximab is essential, and although it seems to be teratogenic during the first trimester,^27^ it may be used with caution in the second and third trimesters, while paying attention to neonatal lymphocytopenia.^26^ Imatinib, a tyrosine kinase inhibitor approved for the management of Philadelphia chromosome-positive chronic myeloid leukemia, crosses the placenta and should not be administered during the first trimester. It has been shown to cause malformations when administered during the first trimester in pregnant women, but appears to be safe during the second and third trimesters.^26^ Angiogenesis inhibitors are teratogenic and have been shown to induce pregnancy loss, skeletal retardations, and fetal growth restriction in animal models due to the crucial role of angiogenesis in the normal development of the placenta and the fetus. Thus, anti-vascular endothelial growth factor and other anti-angiogenic drugs are contraindicated during pregnancy. HER-2-targeted therapy trastuzumab, commonly used for treating HER-2 overexpressing breast cancer, is associated with severe oligo-/anhydramnios and subsequent neonatal respiratory failure due to lung hypoplasia when administered in the second or third trimester, probably due to blockage of the epidermal growth factor receptor-2 expressed in the fetal kidney.^28^ The use of trastuzumab is often delayed until after delivery. Regarding immunotherapy, PD1/PD-L1 and CTLA-4 interactions appear to play key roles in maintaining normal fetal tolerance; not surprisingly, immune checkpoint inhibitors such as anti-PD1/PD-L1 agents have been shown to increase the rate of spontaneous abortions in animals.^29^ However, there are a few case reports of pregnant melanoma patients being treated during the first trimester without miscarriage.^30 31^ Based on the limited evidence available, the use of targeted therapies commonly administered for the treatment of cancer is not supported during pregnancy and should ideally be postponed until after delivery, except for rituximab and imatinib which may be given in the second and third trimesters (Figure 1). Nevertheless, accidental short-term exposure to biologic agents during the first trimester does not justify termination of pregnancy per se.

#### Supportive Treatment

Supportive medication as part of systemic treatment is considered safe for a number of drugs. Anti-emetics may be administered during pregnancy, including metoclopramide and serotonin receptor antagonists,^32^ but safety has not been determined for neurokinin 1 inhibitors.^33^ Use of betamethasone or dexamethasone as premedication is discouraged due to almost 100% placental passage to the fetus, and these are better replaced by steroids that are metabolized in the placenta including methylprednisolone, prednisolone, or hydrocortisone. There is ongoing debate about the use of growth factors, such as granulocyte colony stimulating factor and erythropoietin, although the former has been shown to be safe during pregnancy, permitting dose-dense schedules.^34^

### Radiation Therapy

On account of high fetal radiation dosages and severe or lethal consequences to the fetus, pelvic irradiation should never be performed intentionally during pregnancy (Figure 1).^9^ Generally, radiotherapy for non-pelvic cancers is limited to the first trimester, when the uterus is still at a distance from the irradiation field. The total dose of fetal irradiation comprises a combination of internal scatter, leakage radiation, and external scatter. The internal scatter depends on the source of irradiation and the proximity to the fetus.^9^

Since the impact of irradiation on the fetus depends on gestational age and radiation dose, careful planning in agreement with the patient is essential.^9^ Maternal and fetal consequences of treatment options with and without radiation should be carefully discussed with both the patient and their partner. Using a phantom model, it is recommended that a physicist calculates the fetal radiation dose, and modifications to the treatment plan such as changing the field size, angle, and radiation energy should be considered where possible.

## Obstetric and Perinatal Management

**Figure 2:**
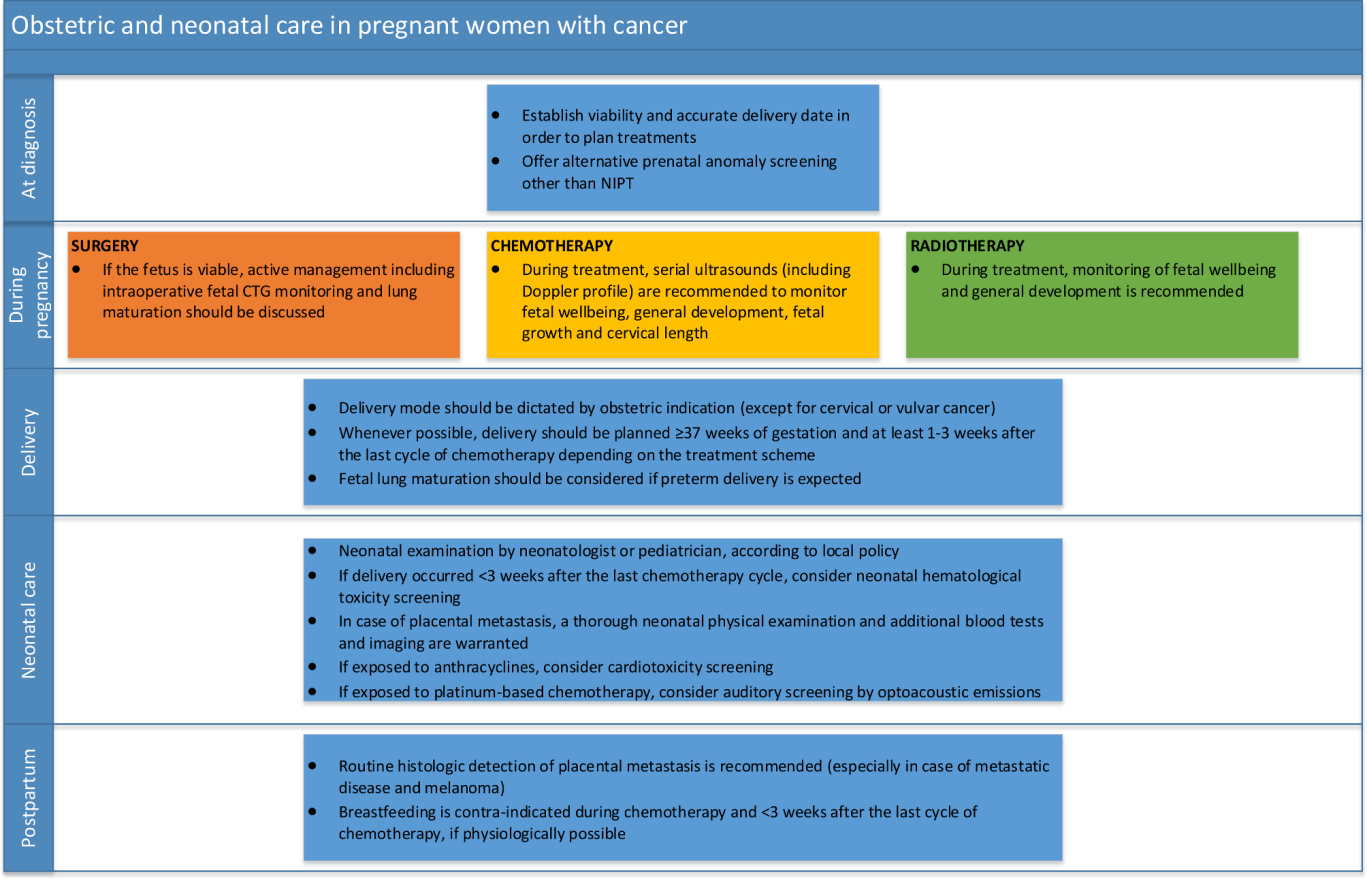
Obstetric and neonatal care in pregnant women with cancer. CTG, cardiotocography; NIPT, non-invasive prenatal testing.

Planning obstetric and perinatal management in women with cancer involves close collaboration between oncologic and obstetric disciplines because of all the different aspects that need to be taken into account (Figure 2). Pregnancy dating should be done early in gestation to permit accurate estimation of gestational age at diagnosis and treatment.

### Obstetric Management

#### Non-Invasive Prenatal Testing

**Figure 3:**
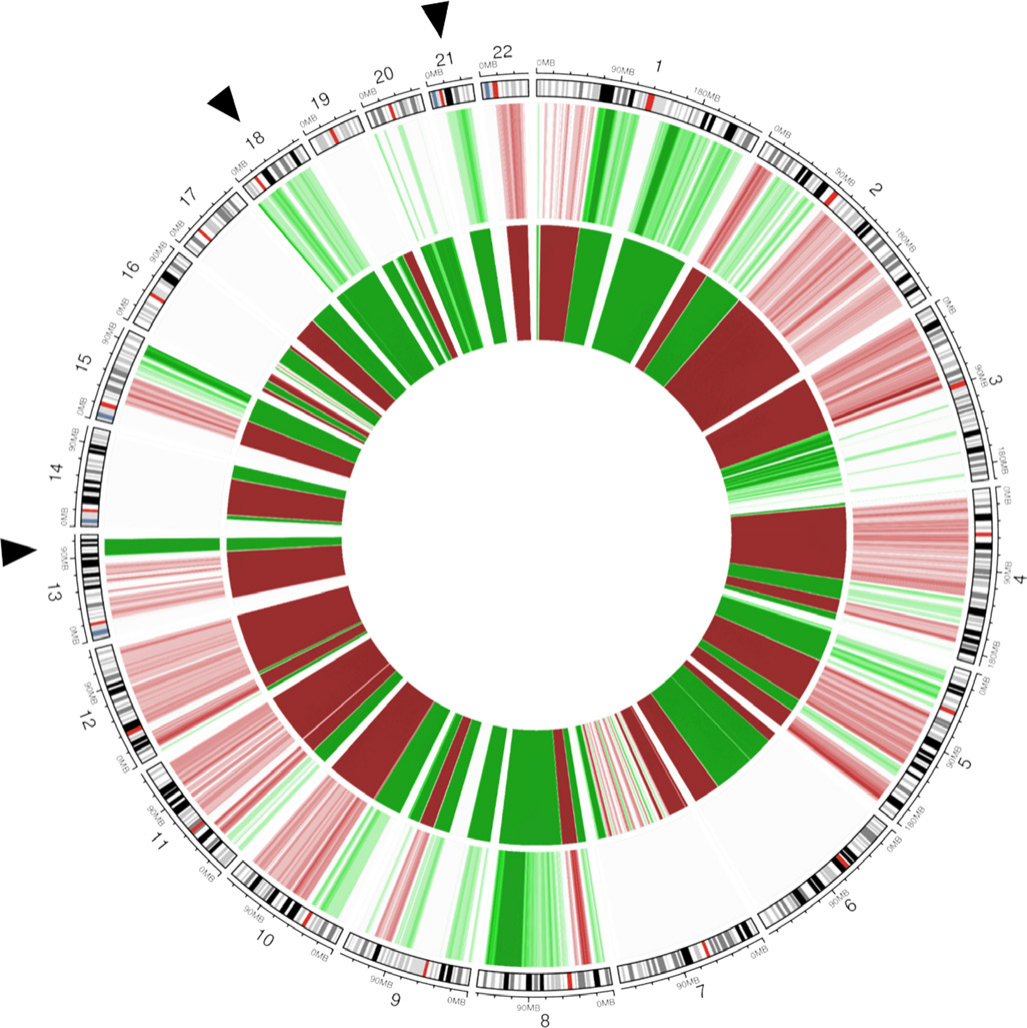
Non-invasive prenatal testing. Circos plot showing chromosomal anomalies detectable in plasma cell-free DNA (cfDNA) and matched tumor biopsy DNA of a pregnant woman who was 8 weeks’ pregnant with a known breast cancer diagnosis. The genomic representation profile of the autosomal chromosomes is shown in clockwise order, aligned with chromosomal ideograms (outer circle). Chromosomal anomalies representing gain are indicated in green; those representing losses are shown in red. Color grades are used to indicate the amplitude of the copy number alterations (CNAs). The middle circle depicts the genome-wide, non-invasive prenatal testing (NIPT) profile in plasma cfDNA with elevated anomalies observed for chromosomes 21, 18, and 13 (indicated by black arrows). On a genome-wide view, (sub)chromosomal imbalances across multiple autosomal chromosomes can be observed. The inner circle shows the copy number profile of matched tumor DNA extracted from formalin-fixed paraffin-embedded tumor biopsy material (whole-genome low-pass sequencing, 0.1 x coverage). Comparison of both profiles reveals that the (sub)chromosomal CNAs and aneuploidies observed in plasma cfDNA are derived from tumor DNA. Adapted with permission from Lenaerts (2019).^38^

Non-invasive prenatal testing is a widely used method to screen for common fetal aneuploidies, primarily designed for trisomy 21 (Down syndrome), trisomy 18 (Edwards syndrome), and trisomy 13 (Patau syndrome). Non-invasive prenatal testing analyzes circulating cell-free DNA (cfDNA) fragments in the blood of pregnant women. A small proportion of these fragments (10%–15% between 10 and 20 weeks of gestation) originates from placental cells, and hence represents fetal DNA; but as the majority of cfDNA is of maternal origin, it may also detect maternal chromosomal abnormalities (Figure 3).^35^ Occult maternal malignancies have been identified as an incidental finding following a deviating non-invasive prenatal testing result not being consistent with the fetal genetic constitution.^36^ As the use of non-invasive prenatal testing is increasingly being expanded to low-risk pregnancies – in some countries it is even offered as a first-tier test to all pregnant women – and its scope is being broadened beyond aneuploidy screening, more discordant results are expected to be identified. When a non-invasive prenatal testing result is suggestive of a maternal cancer, the result should be confirmed by a second non-invasive prenatal test and comparison of the result with the chromosomal profile in maternal tissue.^2^ A multidisciplinary team of specialists is advised to be involved in the diagnostic workup and adequate counseling of the pregnant woman throughout the process.^37^ As a consequence, non-invasive prenatal testing in women with a known cancer diagnosis is non-informative and alternative prenatal screening testing should be offered.^38^

#### Obstetric Ultrasound

Available large case series reveal that neonates prenatally exposed to chemotherapy are at risk of being born small for gestational age, defined as a customized birth weight percentile below 10.^5^ Besides constitutionally small neonates, explained by inherited factors, this definition also incorporates fetuses that did not reach their growth potential and are pathologically growth-restricted. In the pregnant cancer population, risk factors for impaired fetal growth such as maternal age, cancer (especially hematologic cancers), poor general health, malnutrition, and treatment-related stress are common. Whether the chosen regimen of cytotoxic drugs during pregnancy affects the neonatal growth potential is uncertain. Growth-restricted neonates are at risk for adverse outcomes in the short and long term.^39^ In order to detect fetal growth restriction, 2-weekly prenatal ultrasound to monitor fetal growth and amount of amniotic fluid during the course of antenatal chemotherapy is recommended.^21^ This prenatal information will inform obstetricians on fetal well-being and enable them to intervene at signs of fetal compromise. A cardiotocogram can provide additional information. Because chemotherapy, especially platinum and non-platinum alkylating agents, is associated with preterm contractions and delivery,^5^ expert advice includes regular cervical length monitoring (2–4-weekly) in every pregnant patient receiving antenatal chemotherapy.^21^ Theoretically, preterm labor might be explained by stress-related activation of the maternal hypothalamic–pituitary–adrenal axis or chemotherapy-induced apoptosis in fetal membranes causing preterm membrane rupturing. Physicians should be aware of preterm contractions following chemotherapy with a low threshold for admission for observation and administration of intravenous fluids and tocolytics.

### Perinatal Management

#### Delivery

Delivery in oncologic patients is usually planned in order to minimize the treatment-free interval and not impair maternal outcome. Whenever possible, delivery should be planned after 37 weeks of gestation in order to avoid prematurity-related neonatal complications and long-term impairment.^4^ Cesarean section rate in pregnant cancer patients is observed to be over 30%, which is higher compared with the reported worldwide rate of 21%.^40^ This higher rate is partly explained by the high proportion of preterm deliveries and the desire for a planned and controlled delivery in oncologic patients that are subject to psychologic stress and physical exhaustion. Unless obstetrically contraindicated, a vaginal delivery is desired with fewer neonatal and maternal complications. Vaginal delivery is contraindicated for most cervical and vulvar cancers because of the potential for implantation of cancer cells in the vaginal tear/episiotomy site.^21^

Delivery within 3 weeks of chemotherapy regimens, or shorter intervals in cases of 2-weekly or weekly schemes, should be avoided. This safety window allows time for the placenta to metabolize the chemotherapy (especially important for the preterm infant) and for resolution of any myelosuppression on the part of mother and fetus. A report on 49 neonates prenatally exposed to chemotherapy within 4 weeks of birth reported a 33% incidence of transient neutropenia^41^; La Nasa et al reported a 20% incidence of neutropenia at birth in neonates delivered 22–28 days after chemotherapy.^42^ In contrast, 3/54 neonates (5.5%) prenatally exposed to chemotherapy more than 4 weeks before delivery were born with neutropenia. A neonatal blood test after birth should identify risks for nosocomial infections.

#### Placental Examination

Reliable incidence rates of metastatic involvement of the placenta in women with cancer are lacking, as routine histologic detection of placental metastasis is not always performed. Based on approximately 100 published cases, placental metastasis is rare, but may occur in women with malignant melanoma and metastatic disease of any cancer type.^43^ Although even rarer, it is often the only indication for early detection of neonatal metastasis (17% in the group of placental involvement).^44^ Neonatal/fetal metastases have been found mainly in patients with melanoma, and incidentally in lung cancer and leukemia. Placental metastases are mainly located in the intervillous space and to a lesser extent in the villi, probably due to the placental barrier protecting the fetus from hazardous substances in the maternal circulation. Although based on limited evidence, only villous involvement has been described in association with neonatal metastasis. To this end, histologic examination of the placenta is crucial and recommended for the detection of microscopic placental metastasis and the identification of potential fetal involvement, especially in women with melanoma or with advanced disease.

As mentioned earlier, fetal growth restriction is a well-known obstetric complication in pregnancies complicated by cancer.^5^ Uteroplacental vascular insufficiency and the subsequent impact on placental development accounts for the majority of fetal growth restriction in the non-cancer population.^45^ A direct damaging effect of chemotherapy to the placenta has not been identified yet, but is strongly suspected, as placental weight is significantly lower after maternal chemotherapy treatment.^46^ Placental examination in women with cancer, especially those with fetal growth restriction, will provide more insight into cancer treatment-induced placental effects and may influence treatment choice and obstetric management in the future.

#### Lactation

Because of both short- and long-term health benefits, the World Health Organization (WHO) recommends that infants are exclusively breastfed to 6 months of age, with breastfeeding continuing to be an important part of the diet up to 2 years.^47^ For pregnant cancer patients breastfeeding is often highly desired for emotional bonding and as a healthy compensation after prenatal exposure to cancer treatment. However, in clinical practice nursing is discouraged when cytotoxic treatment continues after delivery. Chemotherapy is able to pass into human milk, and although the oral bioavailability is low, case reports have described neutropenia in infants that were breastfed during cancer treatment.^48^ A safety period of 3 weeks between the last administration of most non-platinum chemotherapeutic agents and nursing is strongly recommended. For platinum derivates, the long half-life is a concern, and a case has been described with detectable platinum levels in breast milk more than 3 weeks after last exposure.^49^ Moreover, both chemotherapy and cancer itself might negatively affect the bacterial and metabolic composition of human milk.^50^ Cancer patients that received chemotherapy report reduced milk production and more difficulties with breastfeeding compared with untreated patients, possibly explained by lobular atrophy with fibrosis of breast tissue following chemotherapy exposure.^51^ For breast cancer patients reduced milk production should be expected from the affected breast after breast conservation therapy. Breastfeeding is not recommended in women with hormone receptor-positive breast cancer due to the necessity of adjuvant endocrine therapy.^52^ Cancer patients that consider breastfeeding should be counseled about the risks of low milk production, breast infections, and the hampering of diagnostic and therapeutic procedures in cases of breast cancer because of breast engorgement.

#### Neonatal and Pediatric Management

Children of mothers with cancer during pregnancy require an individualized approach to postnatal care. The different aspects of neonatal and pediatric care depend on the type of cancer, type of treatment exposure in utero, and the timing thereof. Whether every neonate, independent of treatment exposure, should be examined by a neonatologist or pediatrician is based on local protocols. A neonatal complete blood count test is strongly advised to identify myelosuppression, especially if the last chemotherapy cycle was given less than 3 weeks before delivery. In cases with placental metastasis or a suspicion of neonatal metastasis, a thorough physical examination, additional liver panel, abdominal ultrasound, and thorough skin examination in cases of melanoma, should be performed to detect any neonatal disease. Because of anthracycline-related cardiotoxicity in childhood cancer survivors,^53^ a cardiotoxicity screening including echocardiography is recommended for children exposed to anthracyclines in utero. Children exposed to platinum-based chemotherapeutics should be screened for auditory dysfunction using otoacoustic emissions until the age of 5 years, followed by audiometry at later ages.^21^ Additionally, on indication, a consultation with a physiotherapist for developmental follow-up and a geneticist for screening of genetic predisposition to cancer could be offered. The frequency of further visits depends on the existence of fetal metastasis or abnormalities found during the initial visits and examinations.

## Obstetric, Fetal, and Pediatric Outcome After Oncologic Treatment

### Obstetric and Fetal Outcome

No studies have shown an improved maternal survival with termination of pregnancy; control groups are often lacking and stage of disease is not consistently reported limiting the ability to compare severity of disease between women continuing versus terminating a pregnancy. Termination can be considered in cases of aggressive or advanced cancer in early pregnancy.

It is well established that cytotoxic drugs should be initiated after the vulnerable period of organogenesis, and exposure to chemotherapy during the first trimester of pregnancy is associated with a congenital malformation rate of 10%–20%. Overall, reported rates of minor and major birth defects in population-based studies as well as cohort studies in the pregnant cancer population are similar to what is expected in the general population.^5^

**Figure 4:**
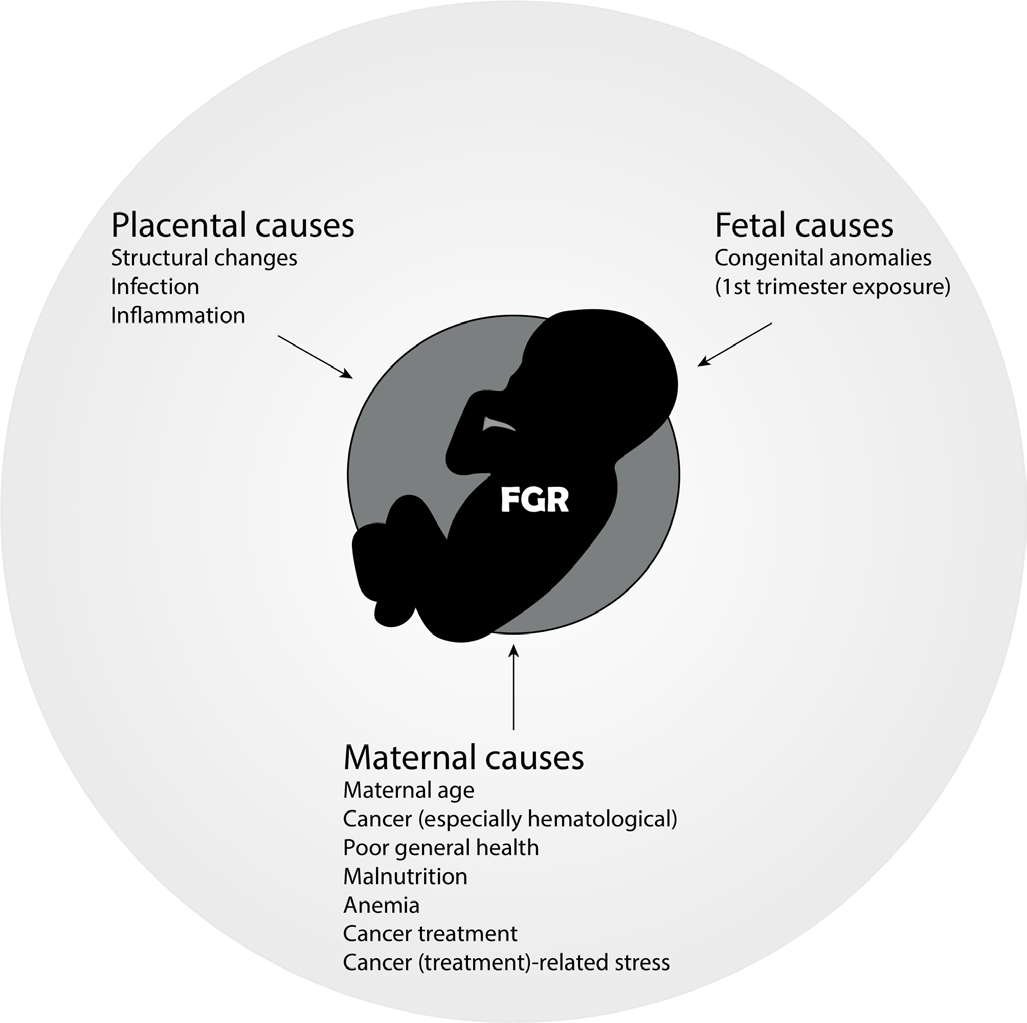
Etiologies of fetal growth restriction (FGR) in women with cancer.

Chemotherapy crosses the placenta and can impact fetal growth, as suggested by several large cohort studies that report a high incidence of small-for-gestational-age or fetal growth restriction, up to 21%, in the pregnant cancer population.^5^ The background incidence of fetal growth restriction varies according to the population, geographic location, and standard growth curves used as reference. In general, 4%–8% of all infants born in developed countries are classified as growth-restricted.^54^ Of note, the underlying maternal disease, cytotoxic treatment during pregnancy, cachexia, cancer-related psychologic stress, and malnutrition are important co-factors in the incidence of fetal growth restriction in pregnancies complicated by maternal cancer (Figure 4). The highest rates of small-for-gestational-age are reported in hematologic and gastrointestinal cancers.^5^ For chemotherapy, de Haan et al reported the highest odds ratio for fetal growth restriction with platinum agents (OR 3.12, 95% CI 1.45 to 6.70) and taxanes (OR 2.07, 95% CI 1.11 to 3.86).

Preterm birth is common, both iatrogenic because of oncologic treatment planning and spontaneous following chemotherapy-provoked preterm contractions. Due to the long-term morbidities for preterm children, avoiding iatrogenic elective preterm births is recommended whenever possible.^55^

Bone marrow toxicity can occur in neonates prenatally exposed to chemotherapy and fetal anemia, secondary to cancer or treatment, can also be detected during pregnancy by Doppler studies.^56^ The combination of poor maternal general health, pancytopenia, systemic illness, oncology-related stress, and cytotoxic drugs might endanger the fetus and the placental function leading to increased risk of intrauterine fetal death, especially in patients with acute leukemia in which intrauterine fetal death is more common compared with other cancers.

Large cohort studies comparing patients that received chemotherapy during pregnancy and patients with a deferral of treatment until after delivery revealed an overall obstetric complication rate of 13%–21%.^57 58^ Premature preterm rupture of membranes and preterm delivery occurred more frequently in the exposed group. Several large population-based studies comparing obstetric outcomes between patients with pregnancy-associated cancer and patients without a cancer diagnosis have been published.^59^ Maternal infections, hypertension, venous-thrombotic events, and postpartum blood transfusions appear to be more common in the pregnant cancer population.^40^ On account of the hypercoagulable state of pregnancy, low molecular weight heparins during pregnancy and, more importantly, until 6 weeks after delivery should be considered.

### Pediatric Outcome

Amant et al^4^ reported on 129 children (median age 22 months) exposed to chemotherapy in utero that showed no significant difference in cognitive outcome and development compared with healthy controls, matched by gestational age at delivery and test age. No significant differences were noted in cognitive skills, academic achievement, or behavioral competence between 35 chemotherapy-exposed children (mean age at evaluation 4.5±3.1 years) and 22 children (4.9±2.6 years) of women with cancer who did not receive chemotherapy prior to delivery.^60^ Normal educational performance was also reported by Aviles et al reporting on 84 children exposed to chemotherapy in utero for maternal hematologic cancer (mean follow-up 18.7 (range 6–29) years).^61^ All children had adequate neurologic and psychologic evaluations. These studies found no adverse effects of chemotherapy on postnatal cognitive function.^4 61^ A recent study of 132 children born to women with cancer, of whom 97 were exposed to chemotherapy, showed normal development at the age of 6 years.^7^ Subtle differences between these children and matched healthy controls were found in verbal intelligence in favor of the latter. Interestingly, in a post hoc analysis, the difference in verbal intelligence was more distinct in the group of children who lost their mother to cancer. Hence, detection of verbal development delays and early psychosocial stimulation to prevent underdevelopment could be considered. In a group of 21 newborns exposed to anthracycline/cyclophosphamide-based chemotherapy in utero, no differences in fetal brain growth were found compared with a group of healthy controls.^62^

The child of a pregnant cancer patient is usually exposed to multiple anticancer drugs, complicating the interpretation of the possible effects of a single drug on their long-term outcome. As previously mentioned, the administration of anthracyclines during childhood is known to cause short- and long-term cardiotoxic effects.^53^ Available cohort studies of children prenatally exposed to chemotherapy are relatively small and represent a maximum follow-up of 20 years. Electrocardiographic results are reassuring.^55^

In childhood cancer survivors, overall hearing loss is seen in up to 48% of children exposed to platinum-based agents, with age being the most important negatively associated risk factor.^63^ In particular, cisplatin is highly associated with ototoxicity. Hearing loss following cisplatin exposure in utero is also reported,^55 64^ but factors such as a middle ear infection, the use of aminoglycosides, and neurodevelopmental problems potentially confound this result.

To date, available long-term outcome studies of children exposed to chemotherapy in utero suggest that chemotherapy is not related to insufficient postnatal growth or impaired cognitive or cardiac function. However, data on long-term outcome are scarce and more studies are needed to provide further insight into long-term safety, including the cancer risk and fertility of the offspring. The International Network on Cancer, Infertility and Pregnancy (INCIP) will continue their work by evaluating the development of children of women with cancer during pregnancy until the age of 18 years and beyond.

## Conclusions

In recent decades, research on the feasibility and safety of oncologic treatment during pregnancy has expanded, resulting in more ongoing pregnancies while treating maternal cancer without delay. As pregnant women with cancer, especially those treated with chemotherapy, face certain obstetric risks, pregnancy monitoring is crucial as is the involvement of perinatologists and neonatologists within a multidisciplinary setting. Moreover, patients and their partners should be supported and encouraged to be actively involved in decision-making regarding oncologic treatment and consequential perinatal decisions. In addition, professional guidance after delivery concerning breastfeeding, physiologic, and psychologic well-being is often highly desired.
